# Chemical immune conization of precancerous cervical lesions awakens immune cells and restores normal HPV negative and abnormal proliferation

**DOI:** 10.3389/fimmu.2023.1259723

**Published:** 2024-01-03

**Authors:** Xueping Liu, Baofa Yu, Feng Gao, Peng Jing, Peicheng Zhang, Guoqin Zheng, Xiaomin Zhang

**Affiliations:** ^1^ Department of Oncology, TaiMei Baofa Cancer Hospital, Dongping, Shandong, China; ^2^ Department of Oncology, Jinan Baofa Cancer Hospital, Jinan, Shandong, China; ^3^ Department of Oncology, Beijing Baofa Cancer Hospital, Beijing, China; ^4^ Department of Internal Medicine, South China Hospital of Shenzhen University, Shenzhen, China; ^5^ Core Lab., Immune Oncology Systems, Inc, San Diego, CA, United States

**Keywords:** cervical cancer, intratumoral chemotherapy, biopsy, H_2_O_2_, penicillin, single-cell RNA sequencing, differentially expressed genes, single-cell copy number variation

## Abstract

**Background:**

Cervical cancer is one of the most common and deadly cancers in women, which is closely linked to the persistent infection of high-risk human papillomavirus (HPV). Current treatment of cervical cancer involves radical hysterectomy, radiotherapy, and chemotherapy or a combination.

**Objective:**

We investigated if hapten-enhanced intratumoral chemotherapy (HEIC) was effective in boosting immunity for effective treatment of precancerous cervical lesions and HPV infection.

**Study design:**

We used single-cell RNA sequencing (scRNA-Seq) to obtain transcriptome profiles of 40,239 cells from biopsies of precancerous cervical lesions from the cervix directly from one patient before the start of HEIC and approximately 1 week after HEIC. The blood samples were taken at the same time as biopsies. We compared the expression characteristics of malignant epithelial cells and immune cells, including epithelial cells, endothelial cells (ECs), fibroblasts, mural cells, T cells, B cells, T and NK neutrophils, mast cells, microparticles (MPs), and platelets, as well as the dynamic changes in cell percentage and cell subtype heterogeneity.

**Results:**

Intratumoral injection of chemotherapy drug plus hapten induces an acute immune response in precancerous cervical lesions with HPV and further awakens immune cells to prevent the abnormal proliferation of the precancerous cells.

**Conclusion:**

HEIC provides a potential treatment method for cervical cancer and HPV infection tailored to each patient’s condition.

## Introduction

Cervical cancer is one of the most common cancers threatening women’s health, which is closely linked to the persistent infection of high-risk human papillomavirus (HPV) (1, 2). The correlation between high-risk HPV infection and precancerous lesions and cervical cancer is extremely high and can be largely preventable. Approximately 90% of cervical cancer occurs in low-income and middle-income countries due to the lack of organized screening and HPV vaccination programs in these countries. In high-income countries, the implementation of screening and vaccination has reduced the incidence rate and mortality of cervical cancer by more than half in the past 30 years.

Treatment of cervical cancer depends on the severity of the disease at the time of diagnosis and the availability of local resources, which may include radical hysterectomy, radiotherapy, and chemotherapy, or a combination of radiotherapy and chemotherapy, which has become a standard of treatment (2). Current treatment of precancerous cervical lesions (cervical intraepithelial neoplasia (CIN)) is very effective, simple, and safe. The entire conversion area of the cervix can be treated through ablation techniques (cryotherapy or thermal ablation) or resection techniques (large ring resection, cold knife conization, or conization). The choice of treatment methods depends on the size and location of the lesion, as well as the type of transformation zone (3).

Due to the lack of intervention in the form of immunotherapy, local recurrence after treatment and persistent HPV positivity are still unresolved issues. The persistence of HPV infection in patients with a high-grade squamous intraepithelial lesion (HSIL) undergoing cervical excision is strongly associated with the recurrence (4).

Inosine pranobex immunotherapy can significantly increase the clearance of viral infection with high-risk genotypes and reduce relapse of HSIL for HPV-positive patients after cervical conization (5). There are several types of immune-related drugs that have been attempted to activate the immune system to improve treatment outcomes. These include immune checkpoint inhibitors, therapeutic vaccines, engineered T cells, and antibody–drug conjugates. Checkpoint inhibitors appear to be the best treatment methods for research, with encouraging Phase II studies in established environments. Vaccines and engineered T cells that use unique immune activation mechanisms are still in the early stages of development (6, 7).

In the current study, we aimed to determine if hapten-enhanced intratumoral chemotherapy (HEIC) was effective in treating precancerous cervical lesions. We hypothesized that HEIC can induce an acute immune response to control both CIN and turning the HPV to negative, a process termed as chemical conization of precancerous cervical lesions. HEIC is used for treating several cancers by hapten-modified tumor-associated antigens or oncogenic proteins expressed in HPV-associated premalignant cervical epithelium (8–10). We carried out biopsies of precancerous cervical lesions using forceps at 12, 3, 6, and 9 o’clock of the cervix directly from one patient before the start of HEIC and approximately 1 week after HEIC. We also took blood samples from the patient before and after HEIC. We then used single-cell RNA sequencing (scRNA-Seq) to obtain transcriptome profiles of 40,239 cells. Through comparative analysis of different samples of CIN and blood samples, we comprehensively described the expression characteristics of malignant epithelial cells and immune cells, including Epithelial Cells, Ecs, Fibroblasts, Mural Cells, Tcells, Bcells, TandNK Neutrophils, Mast Cells, MPs, and Platelets, as well as the dynamic changes in cell percentage and cell subtype heterogeneity. Our results provide evidence that intratumoral co-administration of HEIC induced acute immune response in precancerous cervical lesions to prevent their abnormal proliferation.

## Materials and methods

### Clinical specimens

The patient had a pathological diagnosis and was diagnosed with a clinical stage of HPV-positive cervical intraepithelial neoplasia, CIN 3, in the cervix using the traditional HPV test (7, 8). The patient did not have any other therapy before this study. Before receiving treatment at Beijing Baofa Cancer Hospital, the patient’s physical condition was evaluated and determined to meet the indications for HEIC. This experimental treatment was approved by the hospital ethics committee (TMBF 0010, 2015) in accordance with relevant guidelines and regulations.

After the patient has been prepared for biopsies and blood samples were collected, cleaning and disinfection of the perineum and vagina under general anesthesia were performed, and a disinfecting towel was placed. When precancerous cervical lesions were seen, four small pieces of precancerous cervical lesions (2 mm × 2 mm × 3 mm) were taken at 12, 3, 6, and 9 o’clock of the cervix as an untreated sample for scRNA-Seq analysis. This was followed by intratumoral injection at 12, 3, 6, and 9 o’clock of the cervix of a total of 10 ml containing 1.00 mg/ml adriamycin (Adr), 0.80 mg/ml of cytarabine (Ara-C), 20.0 mg/ml of H_2_O_2_, and 144 mg/ml of penicillin as the hapten (9–11). One week post injection, blood samples were collected again as treated samples, and biopsies of precancerous cervical lesions were carried out again using forceps at 12, 3, 6, and 9 o’clock of the cervix, taking another four small pieces of precancerous cervical lesions (2 mm × 2 mm × 3 mm) as treated samples with blood samples for scRNA-Seq analysis.

### Tissue disassociation and cell collection

After small cervical lesion tissues and blood samples were collected, the fresh tissue samples were immediately stored in the sCelLiVE^®^ Tissue Preservation Solution (Singleron, Nanjing, China) on ice. The tissues were cut into small tissue pieces and were transferred to a 15-ml centrifuge tube, followed by digestion using sCelLiVE^®^ Tissue Dissociation Solution (Singleron) at 37°C for 15 min with shaking. The samples were then filtered using 40-µm sterile strainers and centrifuged at 1,000 rpm at 4°C for 5 min. Next, 2 ml GEXSCOPE^®^ red blood cell lysis bufier (RCLB, Singleron) was added to lyse the red blood cells for 10 min. Finally, the single-cell suspension was collected after re-suspension with phosphate-buffered saline (PBS), and trypan blue (Sigma) staining was used to calculate cell activity and cell count under a microscope.

### Single-cell RNA sequencing

Single-cell suspensions (1~3 × 10^5^ cells/ml) in PBS (HyClone, Logan, UT, USA) were loaded onto a microwell chip using the Singleron Matrix^®^ Single Cell Processing System. Briefly, the scRNA-Seq library was constructed using the GEXSCOPE^®^ Single Cell RNA Library Kits (Singleron). The library was lastly sequenced with 150 bp diluted to 4 nM and paired-end reads on the Illumina HiSeq X platform following an established protocol (12). Sequencing data processing and quality control were performed as described in previous publications (13).

### Differentially expressed gene analysis

To identify differentially expressed genes (DEGs), genes expressed in more than 10% of the cells were selected in both the compared groups of cells and with an average log (fold changes) value greater than 1 as DEGs. The adjusted p-value was calculated using the Benjamini–Hochberg correction. The p-value of 0.05 was used as the criterion to assess the statistical significance.

### Cell type annotation

The cell type identity of each cluster was determined with the expression of canonical markers found in the DEGs using the SynEcoSys database (Singleron Biotechnologies). Heatmaps/dot plots/violin plots displaying the expression of markers used to identify each cell type were generated using the Scanpy built-in functions and ggplot2.

### Single-cell copy number variation analysis

The InferCNV package was used to detect the copy number alterations (CANs) in malignant cells. Non-malignant cells (T and NK cells) were used as control references to estimate the copy number variations (CNVs) of malignant cells. Genes expressed in more than 20 cells were sorted based on their loci on each chromosome. The relative expression values were centered to 1, using a 1.5 standard deviation from the residual-normalized expression values as the ceiling. A slide window size of 101 genes was used to smoothen the relative expression on each chromosome to remove the effect of gene-specific expression.

### Pathway enrichment analysis

To investigate the potential functions of DEGs between clusters, Gene Ontology (GO) and Kyoto Encyclopedia of Genes and Genomes (KEGG) analyses were performed using the “clusterProfiler” R package 3.16.1 (14). The GO gene sets including molecular function (MF), biological process (BP), and cellular component (CC) categories were used as references. Pathways with an adjusted p-value less than 0.05 were considered as significantly enriched.

### Trajectory analysis

Monocle 2 algorithm (15) was used for pseudo-time trajectory analysis, and the dimensionality reduction method used was DDRTree.

### Intratumoral heterogeneity score calculation

The intratumoral heterogeneity (ITH) score was defined as the average Euclidean distance between the individual cells and all other cells in terms of the first 20 principal components derived from the normalized expression levels of highly variable genes. The highly variable gene was identified using the “FindVariableGenes” function in the Seurat package, with default parameters.

### Cell–cell interaction analysis (CellPhoneDB)

Cell–cell interaction (CCI) between B cells, Epithelial cells, Fibroblasts, Mononuclear phagocytes, Mast cells, Neutrophils, and T and NK cells were predicted based on known ligand–receptor pairs by CellPhoneDB v2.1.0 (16–18).

## Results

### Clinical benefit characteristics

In biopsies taken post-treatment, pathological examination confirmed early diagnosis as cervical intraepithelial neoplasia CIN 3. Follow-up examination every 4 weeks after the treatment during a 6-month period, physical examination, and CT of the patient showed no signs of precancerous cervical lesions in the smooth surface of the cervix, and the patient was in good health living a normal life over 1.2 years, during which traditional HPV test continued to yield negative results.

### Landscape of single-cell transcriptome sequencing before and after precancerous lesion treatment

**Figure 1:**
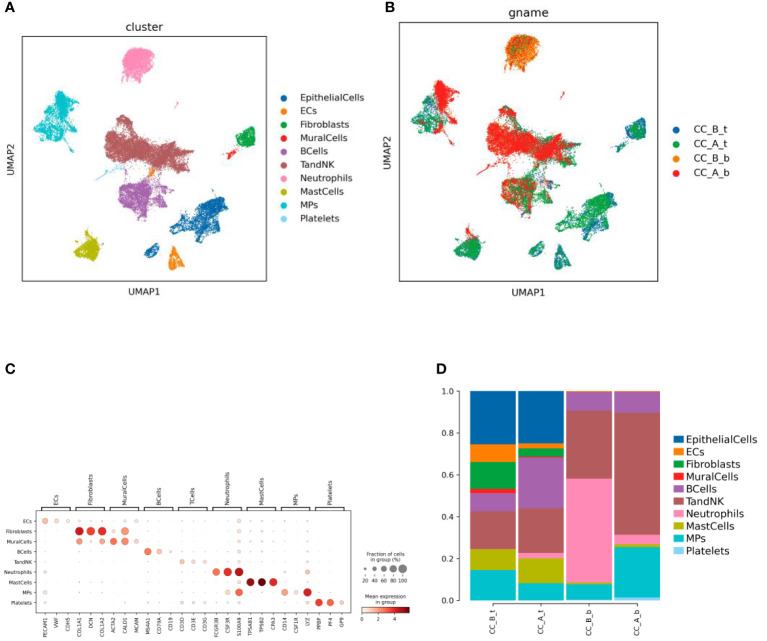
Chemical conization enhances immunotherapy for cervical cancer. **(A)** By using dimensionality reduction clustering to form UMAP cell clustering maps, a total of 10 cell types were obtained, with different colors representing different cell types. **(B)** he distribution of various cell types in tissues and blood before and after treatment, with different colors representing different samples **(C)** Marker gene bubble diagram for each cell type. **(D)** Histogram of cell proportion of each cell type before and after treatment.

Single-cell transcriptome sequencing was performed on two cervical epithelial tissues and their paired peripheral blood mononuclear cell (PBMC) samples before and after treatment. After dimension reduction and clustering, 10 cell types were obtained (**Figure 1A**), including nine in tissues and seven in the blood (**Figure 1B**). Cell types were annotated according to marker genes (**Figure 1C**) of each cell, including EpithelialCells, Ecs, Fibroblasts, MuralCells, Bcells, TandNK Neutrophils, MastCells, MPs, and Platelets.

Analysis of immune cells such as Bcells and TandNK increased in both PBMCs and cervical tissues, while the proportion of stromal cells such as Ecs, Fibroclasts, and MuralCells decreased in tissues before and after treatment. In addition, the proportion of Neutrophils cells in the blood significantly decreased (**Figure 1D**).

### Changes in T cells before and after treatment of precancerous cervical lesions

**Figure 2:**
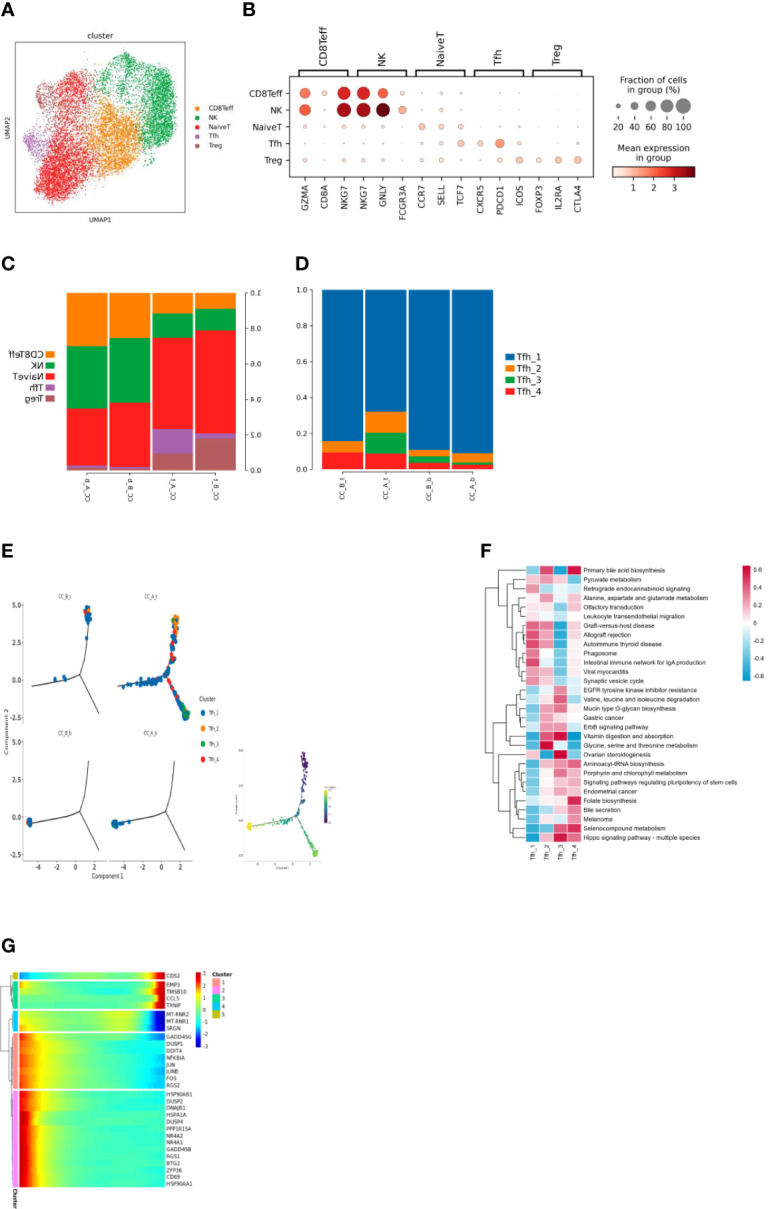
**(A)** T cells were clustered through dimensionality reduction to form UMAP cell clustering maps, obtaining a total of 5 cell types, with different colors representing different cell types. **(B)** Bubble plots of Marker genes in various T cell subpopulations, with color depth representing the average expression of the gene, and circle size representing the percentage of cell expression of the gene in the cell. **(C)** Histogram of the proportion of T cell subpopulations in each sample. **(D)** Histogram of cell proportion of Tfh cell subpopulations in each sample. **(E)** A pseudo time distribution map of a single sample, where one point represents one cell and different colors represent different cell types. **(F)** GSVA analysis of Tfh cell subpopulations, the redder the color, the more enriched the pathway is in the cell. **(G)** Heat map of gene expression changes during the simulated time process, with colors ranging from blue to red representing gene expression from low to high.

Subdividing the T-cell subpopulations yielded a total of five cell types (**Figure 2A**). These cell types were annotated based on the marker genes (**Figure 2B**), including CD8Teff (CD8+ effector T cells), NK (natural killer cells), NaiveT (initial T cells), Tfh (follicular helper T cells), and Treg (regulatory T cells).

Analysis of the proportion of cells before and after treatment showed that after HEIC treatment, the proportion of Tfh cells in the tissue significantly increased. The changes in PBMC samples were not significant (**Figure 2C**).

The increase in follicular helper T cells suggests that treatment of HEIC may stimulate the immune system response and may help combat the development of precancerous cervical lesions. Follicular helper T cells are a specific type of immune cell that can help other immune cells produce stronger immune responses, thereby improving their ability to fight cancer.

Subdivision of Tfh cell subpopulations resulted in four cell types (**Figure 2D**). Analysis of the proportion of each cell before treatment showed that Tfh1 cells were the dominant group, while the richness of Tfh cell subpopulations increased, such as the addition of Tfh3 subpopulations (**Figure 2E**).

The functions of follicular helper T cell subsets show heterogeneity (**Figure 2F**); for example, Tfh1 significantly enriches the phagocytic pathway; Tfh2 significantly enriches the glycine, serine, and threonine metabolic pathways; Tfh3 significantly enhances the biosynthesis and metabolism-related pathways of biomacromolecules such as sugar, amino acid, and vitamin; and Tfh4 significantly stimulates the signal pathway of stem cell pluripotency.

### Time series analysis shows T-cell changes in precancerous cervical lesions before and after treatment

**Figure 3:**
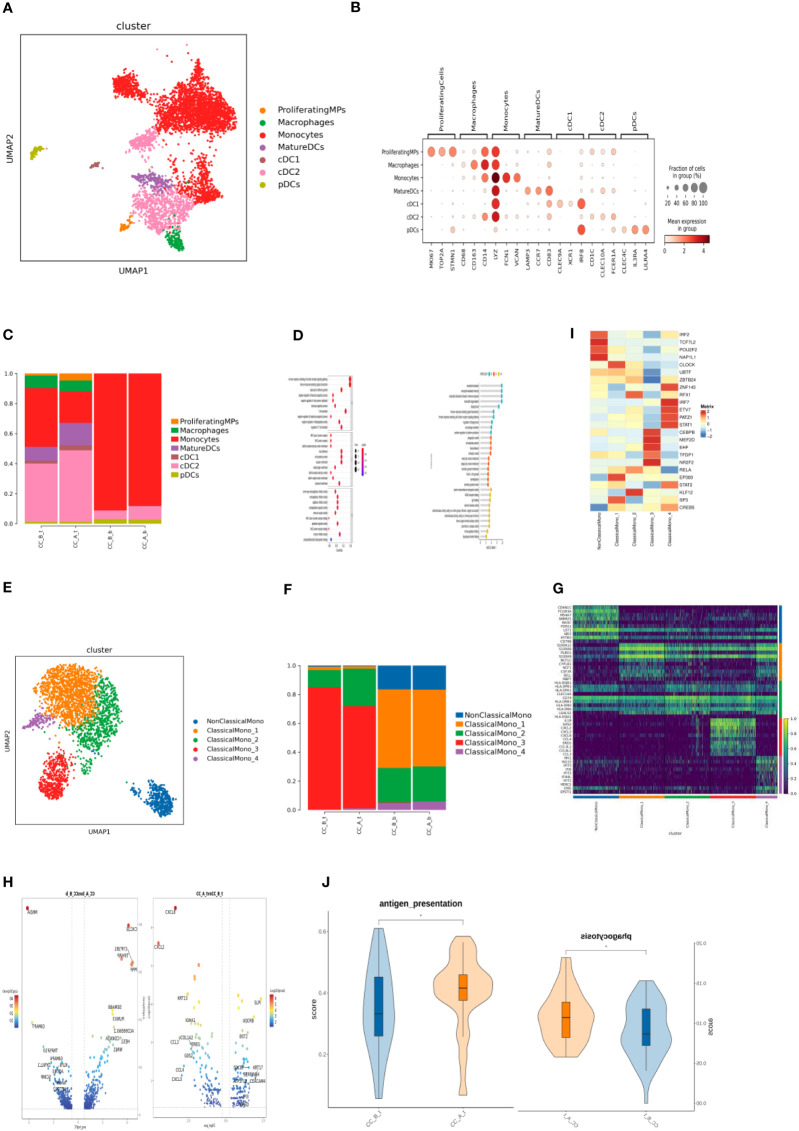
**(A)** MPs cells formed UMAP cell clustering maps through dimensionality reduction clustering, obtaining a total of 7 cell types, with different colors representing different cell types. **(B)** Bubble plots of Marker genes in various MPs cell subpopulations, with color depth representing the average expression of the gene, and circle size representing the percentage of cell expression of the gene in the cell. **(C)** Histogram of cell proportion of MPs cell subpopulations in each sample. **(D)** Bubble diagram (left) and lollipop diagram (right) of GO enrichment analysis results of differentially up-regulated genes of MatureDC cells (left) and cDC2 cells (right) between groups (CC_A_t vs CC_B_t). **(E)** Monocytes cells were clustered through dimensionality reduction to form a UMAP cell clustering map, obtaining a total of 5 subpopulations, with different colors representing different cell types. **(F)** Histogram of the cell proportion of Monocytes cell subpopulations in each sample. **(G)** Top10 differential gene heatmap of Monocytes cell subpopulations. **(H)** Differential gene analysis of ClassicaMono-2 cells between groups, with the left being in the tissue (CC_A_t vs CC_B_t) and the right being in the PBMC sample (CC_A-b vs CC_B-b). **(I)** Transcription factor analysis of various cell subpopulations in Monocytes. **(J)** Antigen presenting ability and cell phagocytosis related gene set scores in tissue samples before and after treatment with ClassicaMono-2 subgroup.

There were differences in the differentiation of Tfh cells in tissues before and after treatment. Before treatment, the cells in the sample were overall located in the early stage of the trajectory. After treatment, the cells were overall located in the early, middle, and late stages (**Figure 3I**).

Before treatment, the main cells present in the tissue sample were Tfh1 cells located at the beginning of differentiation. After treatment, the cells at the beginning of differentiation in the sample were mainly Tfh1. As differentiation progressed, Tfh1 differentiated in two directions: 1) Tfh3 and Tfh4 cells and 2) Tfh1 cells in a differentiated state (**Figures 2G–K**). Changes in gene expression were observed during the differentiation of Tfh1 into Tfh3 and Tfh4 cells (**Figure 2J**): high expression of MT-RNR2, MT-RNR1, and SRGN genes in the middle stage of differentiation. High expression of MT-RNR2 and MT-RNR1 genes indicated that cells were in a differentiation transition state with strong metabolic ability, which changes in gene expression during Tfh1 cell differentiation toward the end of Tfh1 differentiation (**Figure 2J**).

### Changes of MP cells in precancerous cervical lesions before and after treatment

By subdividing T-cell subsets, seven cell types were obtained (**Figure 3A**). According to the marker gene (**Figure 3B**) of each cell, they include proliferating monocyte phagocyte, monocytes, macrophages, mature dendritic cells, type 1 classic dendritic cells (cDC1), type 2 classic dendritic cells (cDC2), and plasma cell-derived dendritic cells (pDCs).

Analysis of the proportion of cells after drug treatment showed that the monocytes in the tissue significantly decreased, while the proportion of cells in MatureDCs and cDC2 increased; the changes in PBMC samples were not significant (**Figure 3C**).

Analysis of intergroup differences in tissues showed that MatureDCs upregulated T-cell activation; MHC II protein complexes, negative regulation of white blood cell apoptosis γ-interferon response, and other related pathways (**Figure 3D**) suggest that MatureDCs can activate and enhance the immune response ability of T cells.

cDC2 upregulates neutrophil activation, macrophage activation, phagocytosis, cytokine production, and RAGE receptor binding-related pathways (**Figure 3E**), suggesting that cDC2 may regulate the activation status of neutrophils, macrophages, and other immune cells, thus participating in the immune response process cooperatively. Five cell types were obtained by subdividing the monocyte cell subpopulations (**Figure 3F**): non-classical monocytes (NonClassicalMono), ClassicalMono_1, ClassicalMono_2, ClassicalMono_3, and ClassicalMono_4.

Analysis of the proportion of cells found that the dominant cell populations in the tissue before treatment were ClassicalMono_2 and ClassicalMono_3, and the proportion of ClassicalMono_3 cells significantly decreased after treatment. In PBMCs, there was little change in cell composition before and after treatment, and the dominant cell population was ClassicalMono_1, which contained more non-classical monocytes than in tissues (**Figure 3G**), indicating heterogeneity between PBMCs and monocytes in tissues.

In tissues and PBMC samples, inflammatory chemokines such as CXCL8, CXCL2, CCL3, CCL4, and CXCL3 were downregulated in the ClassicalMono_2 subgroup, especially CXCL8 (**Figure 3I**), indicating that the inflammatory response may weaken after treatment. The RFX1 transcription factor is specifically overexpressed in the ClassicalMono_2 subgroup (**Figure 3J**).

The ClassicalMono_2 subgroup highly expresses the MHC class II gene (**Figure 3H**). RFX1 transcription factor may regulate the high expression of the MHC class II gene in the ClassicalMono_2 subgroup, thereby enhancing its antigen presentation ability.

### Changes in fibroblast group before and after treatment for precancerous cervical lesions

**Figure 4:**
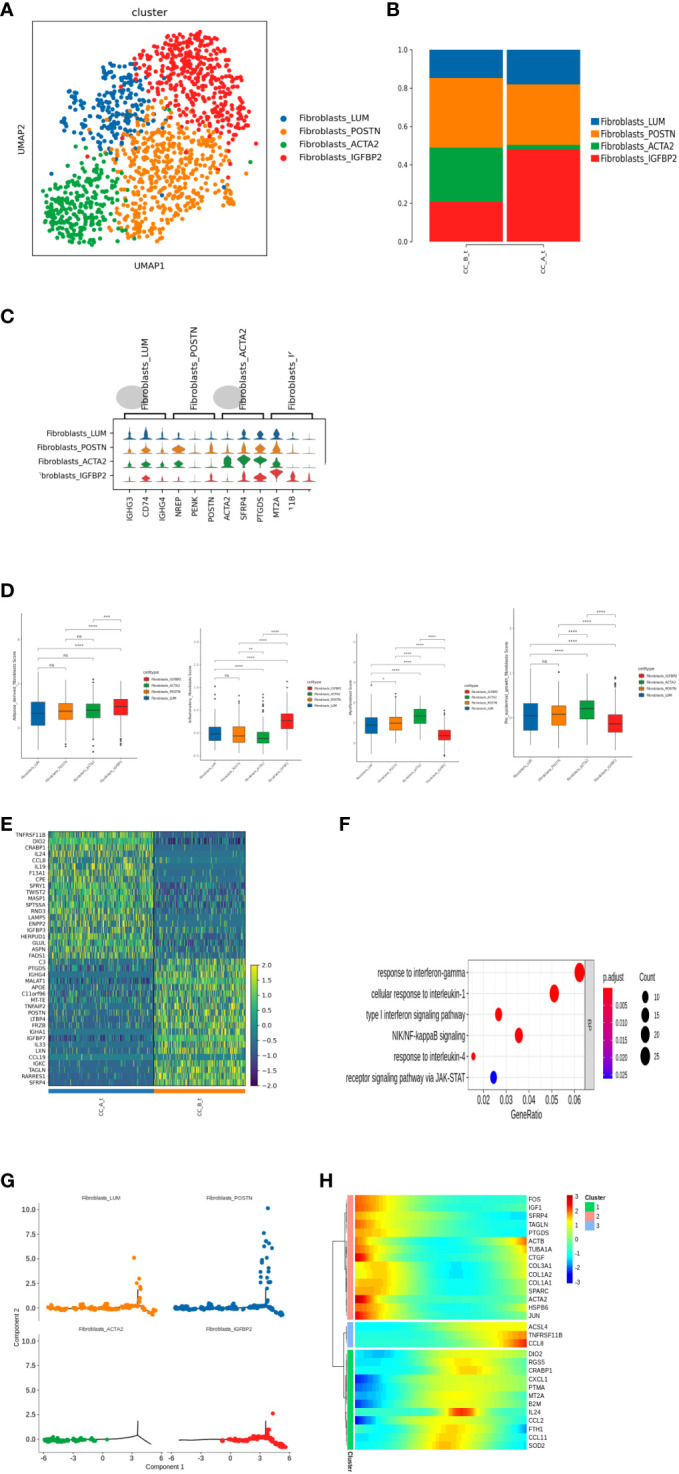
**(A)** Fibroblasts were clustered into UMAP cell clusters through dimensionality reduction, resulting in a total of 4 cell types, with different colors representing different cell types. **(B)** Histogram of cell proportion of fibroblast subpopulations in each sample. **(C)** Top3 differential gene violin map of fibroblast subpopulations. **(D)** The score results of the Ucell gene set of fibroblast subpopulations show the characteristics of adipogenic fibers, inflammatory fibers, myofibroblasts, and epidermal promoting fibroblasts. **(E)** Fibroblasts_ Differential gene expression heatmap of IGFBP2 cell subpopulations between groups. **(F)** Fibroblasts_ GO enrichment analysis of upregulated differentially expressed genes in IGFBP2 cell subpopulations between groups. **(G)** A pseudo time distribution map of a single sample, where one point represents a cell and different colors represent different cell types. The small image shows the pseudo time axis for fibroblast differentiation, and the dark color represents the starting point of differentiation. As the pseudo time progresses, the color from dark to light represents the differentiation from front to back. **(H)** Heat map of gene expression changes during the simulated time process, with colors ranging from blue to red representing gene expression from low to high.

Subdividing the subpopulations of fibroblast cells gave rise to a total of four cell types (**Figure 4A**): Fibroblasts_LUM, Fibroblasts_POSTN, Fibroblasts_ACTA2, and Fibroblasts_IGFBP2 cell subpopulations. Analysis of the proportion of each cell found that after drug treatment, fibroblasts were in the tissue. The proportion of IGFBP2 cells significantly increased. The proportion of cells in the ACTA2 subgroup significantly decreased (**Figure 4B**). Heterogeneity exists between various subpopulations of fibroblasts (**Figure 4C**).

Further analysis of Fibroblasts_Differential gene of IGFBP2 cells between groups found that fibroblasts after treatment of IGFBP2 cells overexpress multiple chemokines and interleukins IL24, IL19, and CCL8 (**Figure 4E**) and upregulate the receptor signaling pathway of JAK-STAT γ-interferon response and NIK/NF-κ inflammatory-related pathways such as B signaling pathway and type I interferon signaling pathway (**Figure 4F**).

Time series analysis showed that there were differences in the differentiation of fibroblasts in tissues before and after treatment. Before treatment, the cells in the sample were overall located in the early and middle stages of the trajectory, while after treatment, the cells were overall located in the middle and late stages (**Figure 4I**).

Before treatment, Fibroblasts_ACTA2 is in the early stage of differentiation, Fibroblasts_IGFBP2 is in the middle and late stages of differentiation, and after treatment, Fibroblasts_ACTA2 cell reduction and Fibroblasts_IGFBP2 increase (**Figures 4G–H**), indicating that fibroblasts after treatment ACTA2 may differentiate into Fibroblasts_IGFBP2 subgroup.

Further score the characteristic gene sets of adipose derived fibroblasts, inflammatory fibroblasts, myofibroblasts, and epidermal promoting fibroblasts for each subgroup of fibroblasts **Figure 4G**).

### Changes in cellular communication before and after treatment of precancerous cervical lesions

**Figure 5:**
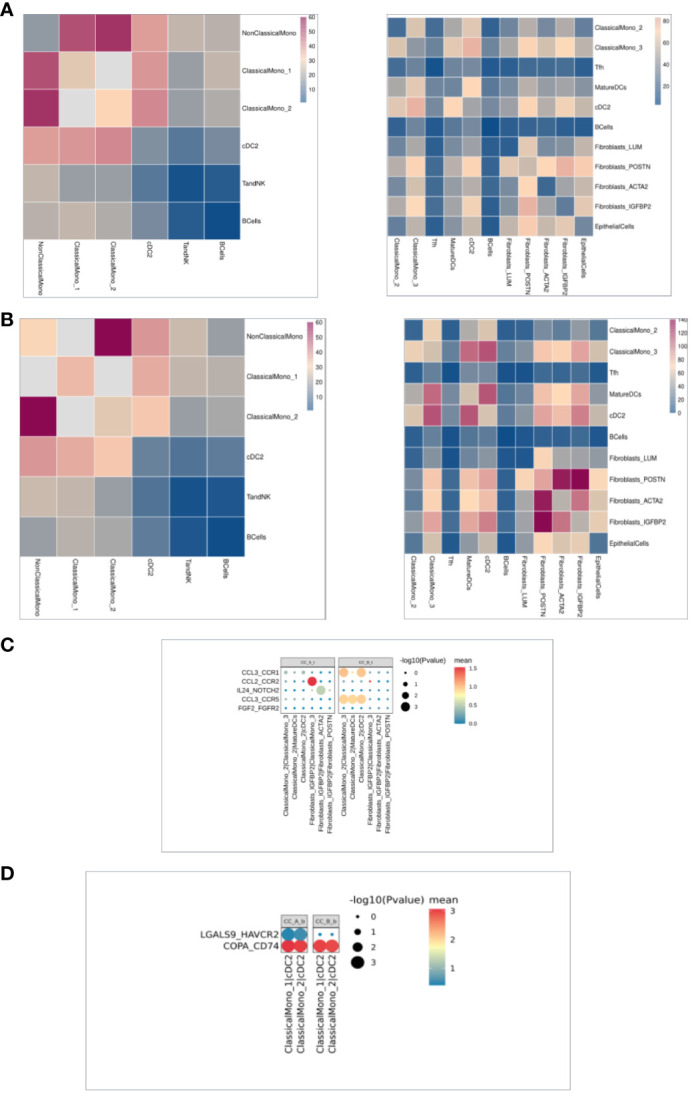
**(A)** The heat map of intercellular communication in the organization, with a redder color indicating stronger intercellular interactions. The left image shows before treatment and the right image shows after treatment. **(B)** The heat map of intercellular communication in PBMC shows that the redder the color, the stronger the intercellular interaction. The left image shows before treatment and the right image shows after treatment. **(C)**. Cellular communication bubble diagram in the organization, with cell pairs on the horizontal axis and ligand pairs on the vertical axis. After treatment on the left and before treatment on the right. **(D)** Cell communication bubble diagram in PBMC, with the horizontal axis representing the cell pair and the vertical axis representing the ligand pair. The left side represents after treatment and the right side represents before treatment.

Cell interaction analysis showed that the signal communication between cells decreased overall in the tissues before and after treatment (**Figure 5A**). Compared to that in PBMC samples before and after treatment, ClassicalMono_1 enhanced communication with other immune cells (**Figure 5B**). Cell interaction analysis in the tissues showed that before and after treatment, ClassicalMono_2/ClassicalMono_3, ClassicalMono_2/MatureDCs, ClassicalMono_2/cDC2, CCL3 between the above cells_CCR1, CCL3. The signal communication between the CCR5 receptor gene pairs is weakened (**Figure 5C**), which is similar to the previous results (**Figure 3I**). On the contrary, Fibroblasts_IGFBP2|Fibroblasts_ACTA2:IL24_NOTCH2 signal enhancement (**Figure 5C**), Fibroblasts (**Figure 4E**). Upregulation of IL24 expression was seen in IGFBP2 cells. Compared to that in PBMC samples before and after treatment, ClassicalMono_1|cDC2 and ClassicalMono_2|cDC2:LGALS9 enhanced signal communication between HAVCR2 (**Figure 5D**).

## Discussion

In this study, we used scRNA-Seq, demonstrated that precancerous cervical lesions (CIN) can be treated with HEIC therapy, and provided evidence supporting that HEIC precancerous cervical lesions with HPV infection induced acute immune response to control the CIN and turned the HPV negative.

Our results showed that cells such as Bcells and TandNK increased in both PBMCs and cervical tissues; the proportion of stromal cells such as Ecs, Fibroblasts, and MuralCells decreased in tissues before and after treatment; and the proportion of Neutrophils cells in the blood significantly decreased. The increase in immune cells may be due to the hapten with drugs killing precancerous cervical lesions with HPV and activating the immune cells, making it effective in activating the immune cells to attack diseased cells. The decrease in stromal cells may be due to the drug affecting the tissue structure and function around the affected cells. These changes may indicate that drug and hapten therapy have had a positive impact on the therapeutic efficacy of CIN 3, consistent in conjunction with clinical outcomes, which are HPV turning negative and cervical surface becoming smooth.

The significant clinical benefit is that one local therapy with hapten and chemotherapy drugs can kill local precancer and hapten modified with tumor-associated antigens, and the major oncogenic protein expressed in HPV-associated precancerous cervical lesions also induces an immune response to fight both precancerous cells and HPV virus.

We used scRNA-Seq and demonstrated that HEIC treatment induces the interaction among Epithelial Cells, Ecs, Fibroblasts, Mural Cells, Tcells, Bcells, TandNK Neutrophils, Mast Cells, MPs, and Platelets, promoting the expression of many genes contributing to the upregulation of immune response in precancerous cervical lesions. Since it is the first attempt to treat precancer positive for HPV, we will need a larger sample size to prove its effectiveness (19).

Detailed subdivision of Tfh cell subpopulations resulted in four cell types with Tfh1 cell in the dominant group enriching the phagocytic pathway while Tfh cell subpopulations increased, as well as the function of follicular T-cell subsets in heterogeneity. This is consistent with the proposed roles for phagocytosis in the degradation of foreign pathogens or cell wastes, which play an important role in immune defense and metabolic regulation (20).

Our results also showed increased expression of CD52, EMP3, TMSB10, CCL5, and TXNIP in the late stage of differentiation; CD52, mainly highly expressed in B cells and T cells, which is an important immune regulatory factor for T-cell activation (21). Overexpression of CD52 leads to increased infiltration of M1 macrophages, monocytes, T-follicle helper cells, and resting memory CD4T cells. CCL5 gene encodes a chemokine ligand 5, which can promote the chemotaxis and aggregation of monocytes, eosinophils, basophils, T cells, natural killer cells, and other immune cells, thus participating in the regulation of immune response and the mediation of inflammatory response (22). TXNIP is thioredoxin that can bind to reactive oxygen species (ROS), avoiding ROS damage to cells and protecting them from oxidative stress (23, 24).

Our analysis reveals that monocytes in the cervical tissue significantly decreased, while the MatureDCs and cDC2 increased in PBMCs. MatureDCs upregulated T-cell activation, MHC II complexes, negative regulation of white blood cell apoptosis γ-interferon response, and other related pathways, suggesting that it enhanced the immune response ability of T cells (21) as well as the γ-interferon response pathway (12, 25).

ClassicalMono_2 and ClassicalMono_3 were the dominant cells in the cervical tissue before treatment, while the ClassicalMono_3 cells significantly decreased after treatment. ClassicalMono_3 overexpresses chemokines (CXCL2, CXCL3, CXCL8, CCL4, CCL3L1, CCL4L2, and CCL3), while ClassicalMono_4 overexpresses ISG56/IFIT1 family genes (ISG15, IFIT3, IFI6, IFIT2, IFI44L, and IFIT1); these genes, stimulated by interferon, play multiple regulatory roles in antiviral immunity and interferon signaling pathways (26).

Treatment decreased inflammatory chemokines such as CXCL2, CCL3, CCL4, and CXCL3, especially CXCL8, in the ClassicalMono_2 subgroup, while the RFX1 (Regulatory Factor X1) transcription factor is specifically overexpressed in the ClassicalMono_2 subgroup. Some target genes of RFX1 are known to include MHC class II genes, which encode important antigen-presenting molecules in the immune system and participate in processes such as antibody-mediated immune responses (27), which is not only for cancer immune reaction but also for HPV immune reaction. Topical application of 2,4-dinitrochlorobenzene (DNCB) as hapten was employed in the immunotherapy of HPV-associated lesions. It was previously found that hapten of DNCB treats skin expressing HPV16. E7 protein, the major oncogenic protein expressed in HPV-associated premalignant cervical epithelium, results in a hyperinflammatory response, with an associated induction of Th2 cytokines and infiltration of myeloid cells producing arginase-1, which also contributes to the hyperinflammation (11).

How does this treatment compare to laparoscopic radical hysterectomy and open approach? Due to laparoscopic radical hysterectomy and open approach treatment of precancerous cervical lesions, the positive endometrial margin is a major risk factor for predicting 5-year recurrence (19). A high risk for having a positive surgical margin, experiencing HPV persistence, positive at the inner margin of the cervix, and positive at the outer margin of the cervix (HR: 6.44 (95%CI: 2.80, 9.65); p < 0.001) was associated with an increased risk of persistence/recurrence. By multivariate analysis, only the inner margin of the cervix was positive, while the outer margin of the cervix was positive (HR: 4.56 (95%CI: 1.23, 7.95); p = 0.021) and was associated with a poorer prognosis. In this high-risk population, a positive cervical margin was the main risk factor for predicting 5-year recurrence (27). This study demonstrates the important role of awakening immune cells in combating HPV-positive precancerous cervical lesions, restoring normal HPV negative and abnormal proliferation, and preventing HPV recovery, which may be a long-term benefit for high-risk people with a positive endocervical margin and experiencing HPV persistence and “low-risk” early-stage cervical cancer (27). It is suggested that immunotherapy should be advanced before or during any treatment to wake up immune cells to recognize tumors or HPV by local administration of drugs and hapten, and PD1 or PD-L1 can be added after waking up immune cells if the patient needs it.

The results presented in our current study have never been demonstrated earlier by a single therapy that can induce an immune response like immunotherapy. Previous studies have reported that the HAVCR2-encoded protein belongs to the immunoglobulin superfamily and TIM family. This gene affects different types of T lymphocytes in the human body and participates in various immune responses, especially in tumor treatment. HAVCR2 can affect tumor growth by regulating T-cell activity and infiltration (28), indicating enhanced immune signal response in PBMCs for whole body immune response.

A significant limitation of the study is the sample size: samples from only one patient were analyzed. Given the significant cost associated with scRNA-Seq, we will seek additional funding support to extend our study to more patient samples.

Our study provides evidence supporting that hapten-mediated local chemotherapy is a safe and effective method because it induces a systematic immunity against both cancer cells and HPV by initiating an immune response from the precancerous cervical lesions to achieve desirable clinical outcomes, which may create a new field of medicine and may be called a chemical immune conization.

## Data availability statement

The original contributions presented in the study are included in the article/supplementary material, further inquiries can be directed to the corresponding author/s.

## Ethics statement

The studies involving humans were approved by Shandong Baofa Cancer Institute. The studies were conducted in accordance with the local legislation and institutional requirements. Written informed consent for participation in this study was provided by the participants’ legal guardians/next of kin. The manuscript presents research on animals that do not require ethical approval for their study. Written informed consent was obtained from the individual(s) for the publication of any potentially identifiable images or data included in this article.

## Author contributions

BY: Investigation, Project administration, Supervision, Writing – original draft. XL: Data curation, Formal Analysis, Resources, Writing – review & editing. FG: Conceptualization, Methodology, Validation, Writing – review & editing. PJ: Methodology, Project administration, Writing – review & editing. PZ: Formal Analysis, Investigation, Methodology, Writing – review & editing. GZ: Formal Analysis, Investigation, Methodology, Writing – original draft. XZ: Data curation, Formal Analysis, Software, Writing – original draft.
